# Pharmacological Activating Transcription Factor 6 Activation Is Beneficial for Liver Retrieval With *ex vivo* Normothermic Mechanical Perfusion From Cardiac Dead Donor Rats

**DOI:** 10.3389/fsurg.2021.665260

**Published:** 2021-06-18

**Authors:** Nuo Cheng, Ji-Hua Shi, Yang Jin, Yuan-Bin Shi, Xu-Dong Liu, Hua-Peng Zhang, Sheng-Li Cao, Han Yang, Wen-Zhi Guo, Shui-Jun Zhang

**Affiliations:** Department of Hepatobiliary and Pancreatic Surgery, Zhengzhou Key Laboratory for HPB Diseases and Organ Transplantation, The First Affiliated Hospital of Zhengzhou University, Zhengzhou University, Zhengzhou, China

**Keywords:** ATF6, donors after cardiac death, normothermic machine perfusion, AA147, ER-stress

## Abstract

**Background:** Normothermic machine perfusion (NMP) could be beneficial for organ retrieval from donors after cardiac death (DCD). Activating transcription factor 6 (ATF6) was recently shown to mitigate liver ischemia/reperfusion injury and confer protection. The aims of this study were to assess the implication of ATF6 in liver retrieval from DCD rat livers with NMP and explore the effect of pharmacologic ATF-6 activation on liver retrieval.

**Methods:** The livers from DCD rats were exposed to 30 min of warm ischemia and 8 h cold preservation followed by 2 h NMP with or without an ATF6 activator in the perfusate. Perfusates and livers were harvested to detect ATF6 expression, liver function, and inflammation.

**Results:** DCD livers with NMP were associated with ATF6 overexpression and activation based on IHC and WB (*P* < 0.05). The ATF6 activator downregulated perfusate aminotransferases, decreased the Suzuki score, downregulated CD68 and MPO based on IHC, induced the expression of cytochrome c in mitochondria and inhibited the expression of cytochrome c in cytoplasm based on WB, reduced TNFα and IL-6 levels based on ELISA, decreased levels of MDA, GSSG and ATP, and increased SOD activity and GSH levels in the perfused livers (*P* < 0.05).

**Conclusion:** ATF6 is important for liver retrieval, and an exogenous ATF6 activator accelerates liver retrieval from DCD rats in an *ex vivo* NMP model.

## Background and Hypothesis

The use of donors after cardiac death (DCD) provides a potential source of vital organs for transplantation to overcome donor shortages. Compared to that with donors after brain death, DCD grafts suffering warm ischemic injury from donor cardiac arrest might be more susceptible with respect to hypoxic cold preservation and reperfusion injury (1). Normothermic machine perfusion (NMP), as a major approach to organ retrieval before transplantation, can recreate the physiological environment of cellular metabolism, oxygenation, and nutrition. Thus, NMP has theoretical advantages over static cold storage (SCS) in benefiting liver transplantation with the utilization of DCDs. Both animal transplant models and clinical data have suggested superiority of the oxygenated NMP against SCS for DCD liver grafts (2, 3). Further, NMP is a promising tool for graft viability evaluation in DBD and DCD environments (4).

Liver ischemia/reperfusion injury, which is involved in cold preservation and NMP of the liver graft, is characterized by a liver inflammatory response. In addition, initial dysfunction in mitochondria and the endoplasmic reticulum due to warm ischemia from DCD might induce a lower threshold of reperfusion injury in liver grafts (5). In the reperfusion stage during NMP, the over-activated acute phase proteins, cytokines, and reactive oxygen species might trigger the unfolded protein response and aggravate hepatic inflammation by activating transcription factor 6 (ATF6)-mediated endoplasmic reticulum (ER) stress (6–8). Oxidative stress characterized by the consumption of Glutathione (GSH) and Adenosine Triphosphate (ATP) and the acute generation of Reactive oxygen species (ROS) is also involved (9).

ATF6 primarily functions to promote protective, adaptive remodeling of cellular physiology, and recovery against ischemia/reperfusion injury (8). The alleviation of ER stress by ATF6 inhibition diminishes the pro-inflammatory effect of liver ischemia, leading to inhibition of the immune response and the protection of livers from ischemia/reperfusion injury (7). The expression and effect of ATF6-mediated chaperones and folding enzymes during DCD liver graft retrieval with NMP remains to be determined. Recently, an ATF6 activator, N-(2-hydroxy-5-methylphenyl)-3-phenylpropanamide (AA147), was found to provide proteostasis-based therapy to ameliorate ischemia/reperfusion injury (10). However, the effect of ATF6 activation on DCD liver grafts under NMP has not been reported previously. Our purposes in the current study were to reveal the expression of ATF6-mediated ER stress during liver graft retrieval from DCD with NMP and to investigate the effect of pharmacologic ATF6 activation on liver retrieval in rats.

## Materials and Methods

### Animals and Study Design

The animal experiment in this study was approved by the Animal Ethics Committee of Zhengzhou University (No.2019-KY-019). Sprague-Dawley male rats weighing 320–350 g were purchased from the Beijing Vital River Laboratory Animal Technology Co., Ltd. (Beijing, China). All rats were housed under specific pathogen-free conditions at room temperature with a 12-h light/dark cycle and were allowed free access to chow and water before surgical procedures. All rats were given pentobarbital (Sinopharm Chemical Reagent Co., Ltd, 0.06 g/kg) via intraperitoneal anesthesia.

To compare the effect of ATF6 activation on graft retrieval with NMP, we designed the following experimental groups: control, *n* = 6, intact fresh livers (non-DCD) without ischemia; SCS, *n* = 6, DCD livers exposed to 30 min of *in situ* warm ischemia and 8 h static cold preservation; NMP, *n* = 6, DCD livers exposed to 30 min of *in situ* warm ischemia and 8 h cold ischemia preservation followed by 2 h NMP; AA147, *n* = 6, DCD livers exposed to 30 min of *in situ* warm ischemia and 8 h cold ischemia preservation followed by 2 h NMP and treatment with the ATF6 activator AA147 (dissolved in 10% dimethyl sulfoxide stock solution); vehicle control, *n* = 6, DCD livers exposed to 30 min of *in situ* warm ischemia and 8 h cold ischemia preservation followed by 2 h NMP with administration of the same amount of dimethyl sulfoxide solution as that in the AA147 group. The detailed grouping information is shown in Table 1.

**Table 1:** Grouping information in the study.

**Group**	**Dispose**			**Number**
	**Warm ischemia**	**SCS**	**NMP**	
Control	(-)	(-)	(-)	6
SCS	30 min	8 h	(-)	6
NMP	30 min	8 h	2 h	6
AA147	30 min	8 h	2 h^*^	6
Vehicle control	30 min	8 h	2 h^†^	6

* *ATF6 activator AA147 and dimethyl sulfoxide solution was used during NMP*.

† *Dimethyl sulfoxide solution was used during NMP*.

### Rat DCD Model and Procurement of Liver Graft

A rat DCD model was employed with the induction of cardiac arrest due to incision of the diaphragm without prior heparinization (2). A volume of 12–15 ml whole blood was withdrawn for perfusate supplementation from the abdominal aorta instantly at the start of cardiac arrest. During warm ischemia, common bile duct, hepatic artery, and portal vein (PV) were isolated and prepared for cannulation by NMP. After 30 min of warm ischemia, *in situ* cannulation of common bile duct, hepatic artery, and PV was accomplished with the appropriate perfusion cannulas (24G, 20G, and 22G IV Catheter System, BD Intima II ^TM^). Liver grafts were flushed with heparin saline at 0~4°C and UW solution through both the hepatic artery and portal vein and then preserved in UW solution at 0~4°C for 8 h.

### *Ex vivo* Rat Liver NMP System and Its Operation

**Figure 1:**
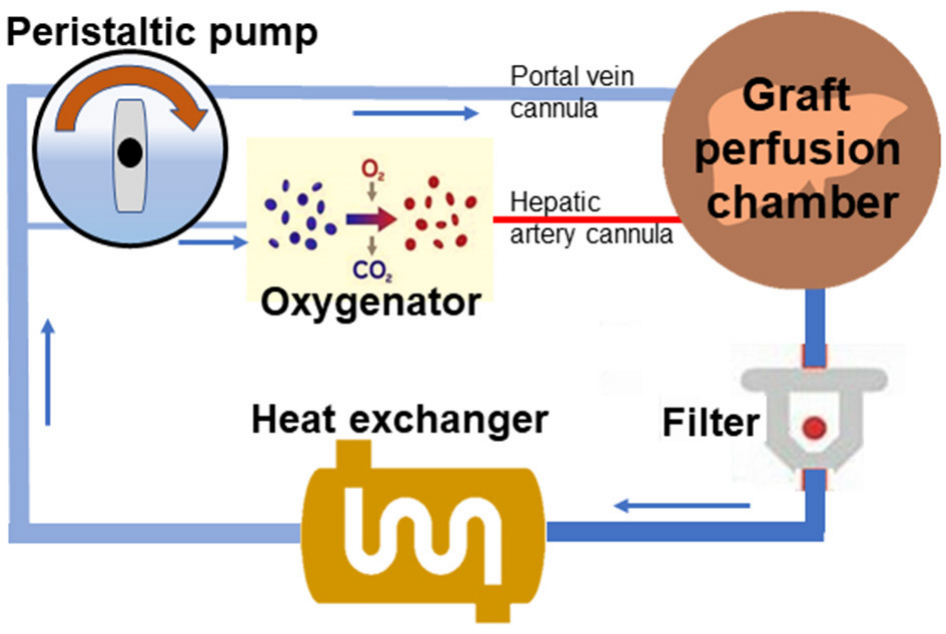
Scheme of the normothermic mechanical perfusion system. The primary circuit of the perfusion system comprises perfusate that could recirculate through a liver perfusion chamber, a blood transfusion filter, a heat exchanger, a peristaltic pump, and a membrane oxygenator with connection by tubing.

The primary circuit of the perfusion system was modified from previous models (2, 11) and comprised perfusate that recirculated via a peristaltic pump through a perfusion chamber, blood transfusion filter with a pore size of 20–40 μm, a heat exchanger, and a membrane oxygenator (Figure 1). The perfusion medium, with a total volume of 36 ml, contained 24 ml whole heparinized blood (collected from two donor rats) supplemented with 10% sodium citrate, 1% penicillin, and streptomycin and 12 ml circuit priming solution with 45% lactated ringer, 5% sodium bicarbonate, and 50% hydroxyethyl starch. Temperature within the system was maintained at 38°C by the heat exchanger and bath thermostat. Two parallel circulations were regulated with a peristaltic pump (BT-100CA, JieHeng Pump Ltd, Chongqing, China) in a PV pressure-dependent manner with a pressure transduction system (BIOPAC systems, Inc. Goleta, USA), which provided the continuous flow of oxygenated perfusate through the hepatic artery and non-oxygenated perfusate through the PV with a flow ratio of 1:3. The oxygenator in circulation of the hepatic artery was regulated with mechanical ventilation (Harvard Inspira Advanced Safety Ventilator, Pressure Controlled MAI 55-7059, Holliston, USA) gassed with a mixture of air and O_2_ to maintain a constant pO_2_ pressure. A hollow fiber dialyzer with a 60-cm^2^ membrane area (Spectrum Labs, Rancho Dominguez, CA) was used. Both the hepatic artery and PV of liver grafts were connected to the perfusion system through perfusion cannulas, and the biliary cannula was connected to an extension tube for external biliary drainage. Liver grafts in the organ perfusion chamber were perfused for 2 h with a constant PV perfusion pressure of 8–0 mmHg and hepatic artery pressure of 90–100 mmHg. The oxygen concentration was adjusted to blood gas values (pO_2_ > 400 mmHg) with an oxygenator. Blood gas analysis and pH were measured every 30 min during perfusion. The portal venous blood flow fluctuated between 5 and 15 ml/min, and the ratio between the portal vein flow and artery flow was 3:1. Initial pH of the perfusate was set at 7.4 (2), and the final pH varied between 7.35 and 7.45. The ATF6 activator, AA147 (Cat. No.6759/5, Tocris Bioscience, Minneapolis, USA), was dissolved in perfusate at a final concentration of 5 mg/l.

### Sample Collection and Analysis

Perfusate samples for blood gas analyses were withdrawn from the cannula placed in hepatic artery at the three-way stopcock and analyzed using an automatic blood gas analyzer (GEM Premier 4000, Instrumentation Laboratory Co., Lexington, USA). Perfusate samples were taken from the PV cannula for measurements of alanine aminotransferase (ALT) (c009-2-1,Nanjing Jiancheng Bioengineering Institute, CHINA) and aspartate aminotransferase (AST) (c010-2-1, Nanjing Jiancheng Bioengineering Institute, CHINA) using standard biochemical methods. Liver biopsies harvested from the perfused liver were immediately snap frozen for mRNA and protein detection, fixed with 10% buffered formalin for morphological evaluation by hematoxylin and eosin (HE) staining, and immunohistochemistry (IHC) and kept at 0~4°C for assessment of superoxide dismutase (SOD) activity, malondialdehyde (MDA) content, ATP content, GSH content, oxidized glutathione (GSSG) content and the level of tumor necrosis factor α (TNF-α) and interleukin 6 (*IL-6*).

### Gene Expression Analysis and Real-Time Quantitative Polymerase Chain Reaction (RT-qPCR)

Total RNA was isolated from liver tissue samples or cells by homogenization in chaotropic salts and subsequent ion exchange chromatography using RNAiso reagent (Takara, Shiga, Japan), and the concentration of RNA was measured using Nanodrop2000 (Thermo Fischer, Waltham, USA). RT-qPCR was performed as previously described (12). Total RNA was reverse transcribed with the PrimeScript® RT reagent Kit (Takara, Shiga, Japan) and subsequently RT-qPCR of each sample was run in triplicate with TB Green™ Premix Ex Taq ™ II (Takara, Shiga, Japan) using QuantStudio5 and software (Applied Biosystems, Foster City, CA) according to the manufacturer's instructions. Gene expression was determined using GAPDH as housekeeping gene. Specific primers for RT-qPCR used in the study (Table 2) were designed and synthesized by Invitrogen (Waltham, USA).

**Table 2:** Primer sequences for RT-PCR in the study.

**RT-PCR primers**	**Sequences**	**Species**
ATF6 forward	GAACTTCGAGGCTGGGTTCA	Rat
ATF6 reverse	AACTTCCAGGCGAAGCGTAA	
PPARα forward	TGCGACATCATGGAACCCAA	Rat
PPARα reverse	CACAATCCCCTCCTGCAACT	
RCAN1 forward	GCCAGAGTACACACCCATCC	Rat
RCAN1 reverse	GGACATAGACTGAGGTGCGG	
IL6 forward	CATTCTGTCTCGAGCCCACC	Rat
IL6 reverse	AGTCTCCTCTCCGGACTTGT	
TNFα forward	AAGCTGTCTTCAGGCCAACA	Rat
TNFα reverse	CCCGTAGGGCGATTACAGTC	
GAPDH forward	AGTGCCAGCCTCGTCTCATA	Rat
GAPDH reverse	TGAACTTGCCGTGGGTAGAG	

### HE Staining and IHC

Liver tissue were fixed in 4% paraformaldehyde in phosphate-buffered, embedded in paraffin wax, and stored at 4°C. HE staining and IHC were performed as before (13, 14). Histological analysis and staining index were evaluated according to Suzuki histological criteria by pathologist (13, 15). IHC analysis was conducted with primary antibodies against ATF6 (1:50, 24169-1-AP, Proteintech, Wuhan, China), CD68 (1:100, 28058-1-AP, Proteintech, Wuhan, China), myeloperoxidase (MPO, 1:100, 22225-1-AP,Proteintech, Wuhan, China) using rabbit or mouse specific IHC detection reagents (1:100; SPN-9001/SPN-9002, Beijing Zhongshan Golden Bridge Biotechnology Co., Ltd.).

### Western Blot

Protein extracts of liver tissue were prepared and separated by SDS-polyacrylamide gel electrophoresis (SDS-PAGE), transferred to polyvinylidene difluoride (PVDF) membrane by electroblotting, and processed for Western blot analysis as previously described (12). Primary antibodies used were against ATF6(1:500, 24169-1-AP,Proteintech, Wuhan, China), peroxisome proliferator-activated receptor α (PPARα, 1:1,000, 11587-1-AP,Proteintech, Wuhan, China), regulator of calcineurin 1 (RCAN1, 1:1000, ab185931, Abcam, Cambridge, UK), cytochrome c(1:1,000,11940s, Cell Signaling Technology, America), Cytochrome c oxidase subunit 4(cox4,1:1,000, A01060A488Abkkine,Wuhan,China),GAPDH (1:5,000, 60004-1-Ig, Proteintech, Wuhan, China). Immunoreactivities were visualized by secondary horseradish peroxidase-conjugated rabbit (1:2,000, SA00001-2,Proteintech, Wuhan, China), mouse (1:2,000, SA00001-1, Proteintech, Wuhan, China**)**, and the ECL Western Blotting Substrate (Solarbio Life Sciences, Beijing, China) according to the manufacturer's instruction of KPL Protein Detector Western Blot Kit.

### Determination of SOD Activity, Content of ATP, GSH, GSSG, and MDA, and Level of TNF-α and IL-6

Detection of the SOD activity, content of ATP, GSH, GSSG, and MDA was quantified by thiobarbituric acid assay, colorimetric method, Microplate method and WST-8 according to the manufacturer's instructions (S0131 and S0103, Beyotime Biotechnology, Haimen, China; A095-1-1 and A061-1, Nanjing Jiancheng Bioengineering Institute, China). Results were obtained using a Multiscan FC plate reader with SkanIt software (Thermo Scientific).

Inflammatory cytokine (TNF-α and IL-6) levels in the liver were measured by ELISA according to the manufacturer's protocols (KE20001, Proteintech, Wuhan, China; EK0412, Boster Biological Technology, Wuhan, China).

### Statistical Analysis

Values are given as means with standard deviation (SD). Differences between two groups were analyzed by the two*-*tailed unpaired Student'*s* test and differences of more than two groups by one-way ANOVA. The statistical tests were employed by using SPSS version 21.0 (IBM, Armonk, New York, USA), and the results for *t* test and ANOVA were denoted as *t* and *F*. A probability level of <5 per cent (*P* < 0.05) was considered statistically significant.

## Results

### NMP Improves the DCD Liver Manifestations

With the *ex vivo* NMP system in the NMP and AA147 groups, 18 rat livers from DCD were successfully perfused. Through gross observation, liver swelling and congestion with an uneven texture and rounded edges on the surface of the livers from DCD rats were visible after warm ischemia and cold preservation. We noted a gradual decrease in the manifestations of liver swelling and congestion during the perfusion process. At the end of the perfusion period, these manifestations disappeared (Supplementary Figure 1).

### NMP Induces ATF6 Expression and Activation in Retrieval DCD Liver

**Figure 2:**
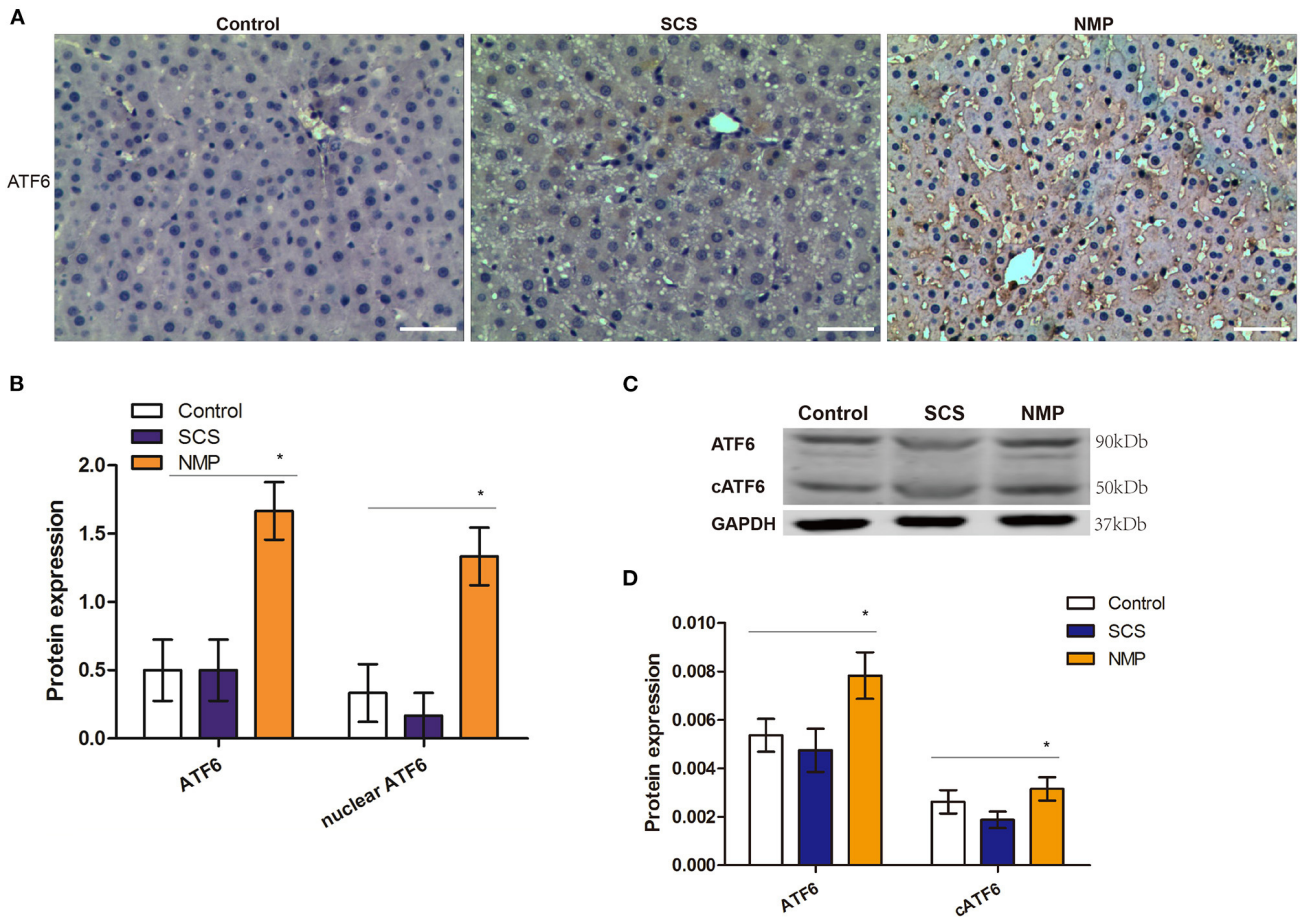
Expression and activation of ATF6 in rat livers of donation from cardiac death (DCD) following static cold preservation (SCS) and normothermic machine perfusion (NMP). **(A)** Immunostaining of ATF6 (haematoxylin counterstaining, original magnification ×200, scale bars 50 μm); **(B)** Semi-quantification staining of total and nuclear ATF6 by immunohistochemistry staining is expressed as means ±SD. **(C)** Expression of ATF6 and cleaved ATF6 by WB. **(D)** Semi-quantification staining of ATF6 and cleaved ATF6 (cATF6) by WB (*Denotes significantly different vs. control and SCS, *P* < 0.05; *n* = 6).

To determine whether the ATF6 pathway was activated during liver graft retrieval, we assessed the expression of ATF6 in the liver tissues by IHC analysis. We found that both cytoplasmic and nuclear expression of ATF6 could be detected in the rat liver cells of the NMP group, indicating that NMP induced both the expression and activation of this marker (Figure 2A). Total and nuclear ATF6 increased >3-fold in rat liver tissues of the NMP group, in comparison with levels in the control and SCS groups (*F* = 6.667 and 12.955, *P* = 0.008 and 0.001, respectively; Figure 2B). Consistent with this finding, full-length ATF6 and cleaved ATF6 (cATF6) in the livers of the NMP group was found to be significantly higher than that in control and SCS groups, as determined by WB (*F* = 3.819 and 4.144, *P* = 0.031 and 0.019, respectively; Figures 2C,D). These results indicated that the NMP process induced the expression and activation ATF6 in the liver cells of the retrieved DCD liver graft.

### AA147 Activates ATF6 in DCD Livers Under NMP

**Figure 3:**
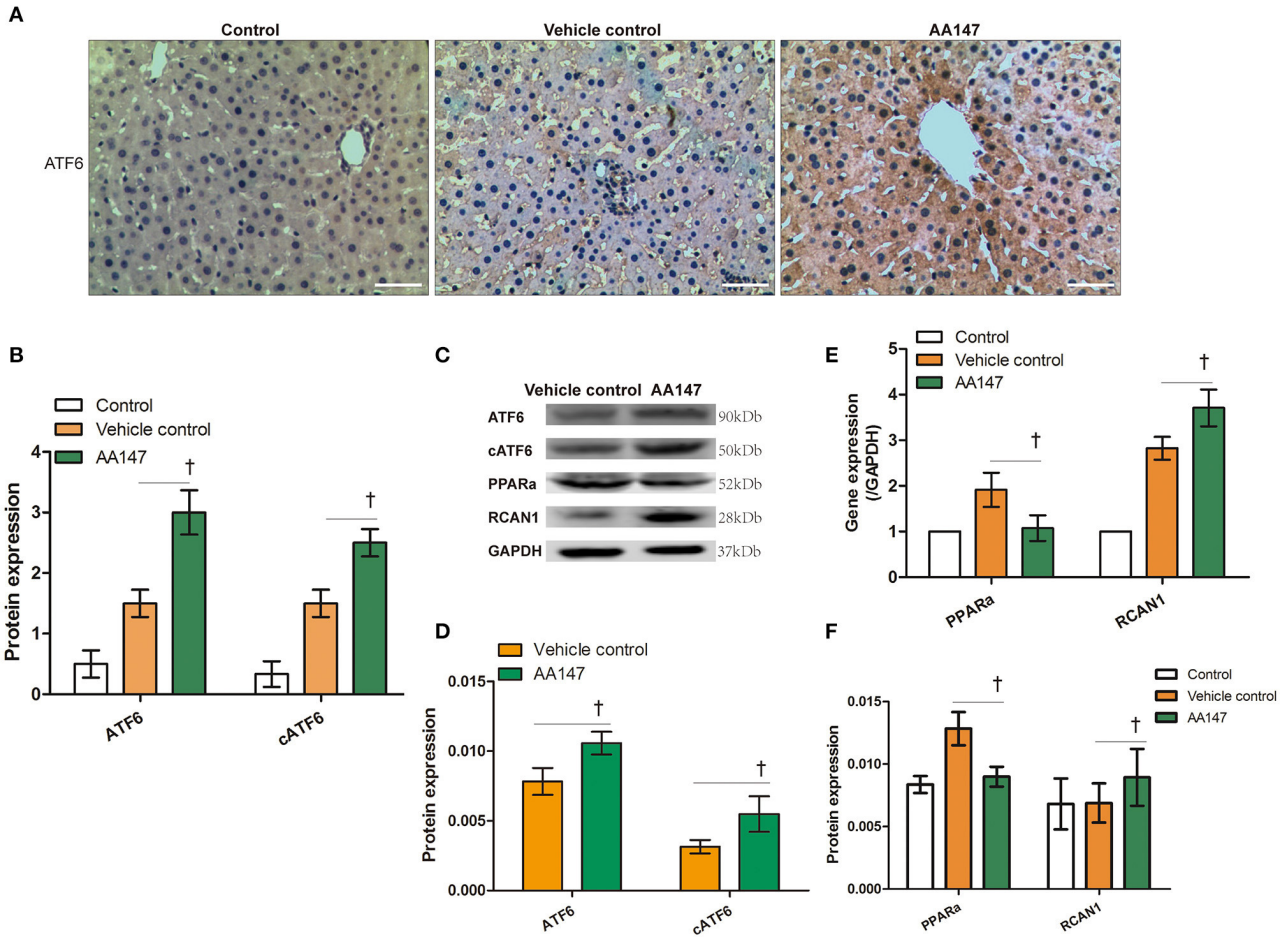
Expression of ATF6 with AA147 administration in livers donation from donation of cardiac death following static cold preservation (SCS) and normothermic machine perfusion (NMP). **(A)** Immunochemistry staining of ATF6 (haematoxylin counterstaining, original magnification ×200, scale bars 50 μm); **(B)** Semi-quantification staining of ATF6 and nuclear ATF6 (cATF6) by IHC. **(C)** Expression of ATF6, cleaved ATF6 (cATF6), PPARα, RCAN1 by WB; **(D)** Semi-quantification staining of ATF6 and cleaved ATF6 (cATF6) by WB; **(E)** Relative changes of ATF6 regulated PPARα and RCAN1 genes by RT-qPCR; **(F)** Semi-quantification staining of PPARα and RCAN1 genes by WB (^†^Denotes significantly different between vehicle control group and AA147 group, *P* < 0.05; *n* = 6).

Pharmacological activation of ATF6 was shown to have a protective role in multiple ischemia/reperfusion models (10). To determine if ATF6 activation has a protective function in DCD liver retrieval, a potent ATF6 activator, AA147, was administered together with NMP treatment. The effect of AA147 on intrahepatic ATF6 was first determined by IHC (Figure 3A). By IHC, we found that both cytoplasmic and nuclear ATF6 expression in the liver tissue of the AA147-treated group were significantly higher than those in the vehicle control group (*t* = 3.503 and 3.162, *P* = 0.006 and 0.010, respectively; Figure 3B). Consistently, the expression of cATF6 in the AA147-treated group was significantly higher than that in the vehicle control group, as determined by WB (*t* = 2.611, *P* = 0.026, respectively; Figures 3C,D).

To further confirm that ATF6 was activated, we determined the mRNA levels of the ATF6-regulated markers *PPARA* and *RCAN1*, for which expression is suppressed and activated by ATF6, respectively (16, 17). The RT-qPCR analysis showed that *PPAR*α expression was inhibited, whereas *RCAN1* expression was induced, in rat liver tissues of the AA147-treated group, compared to levels in the vehicle control group (*t* = 2.242 and 2.447, *P* = 0.042 and 0.041, respectively; Figure 3E). This observation was confirmed by WB analysis (*t* = 2.754 and 2.971, *P* = 0.041 and 0.025, respectively; Figures 3C,F). Taken together, these data demonstrated that the administration of AA147 to the DCD liver under NMP could further enhance ATF6 activation and its transcriptional program, as describe previously (18).

### The ATF6 Activator Alleviates Inflammation Injury and Improves Functions of DCD Livers Under NMP

**Figure 4:**
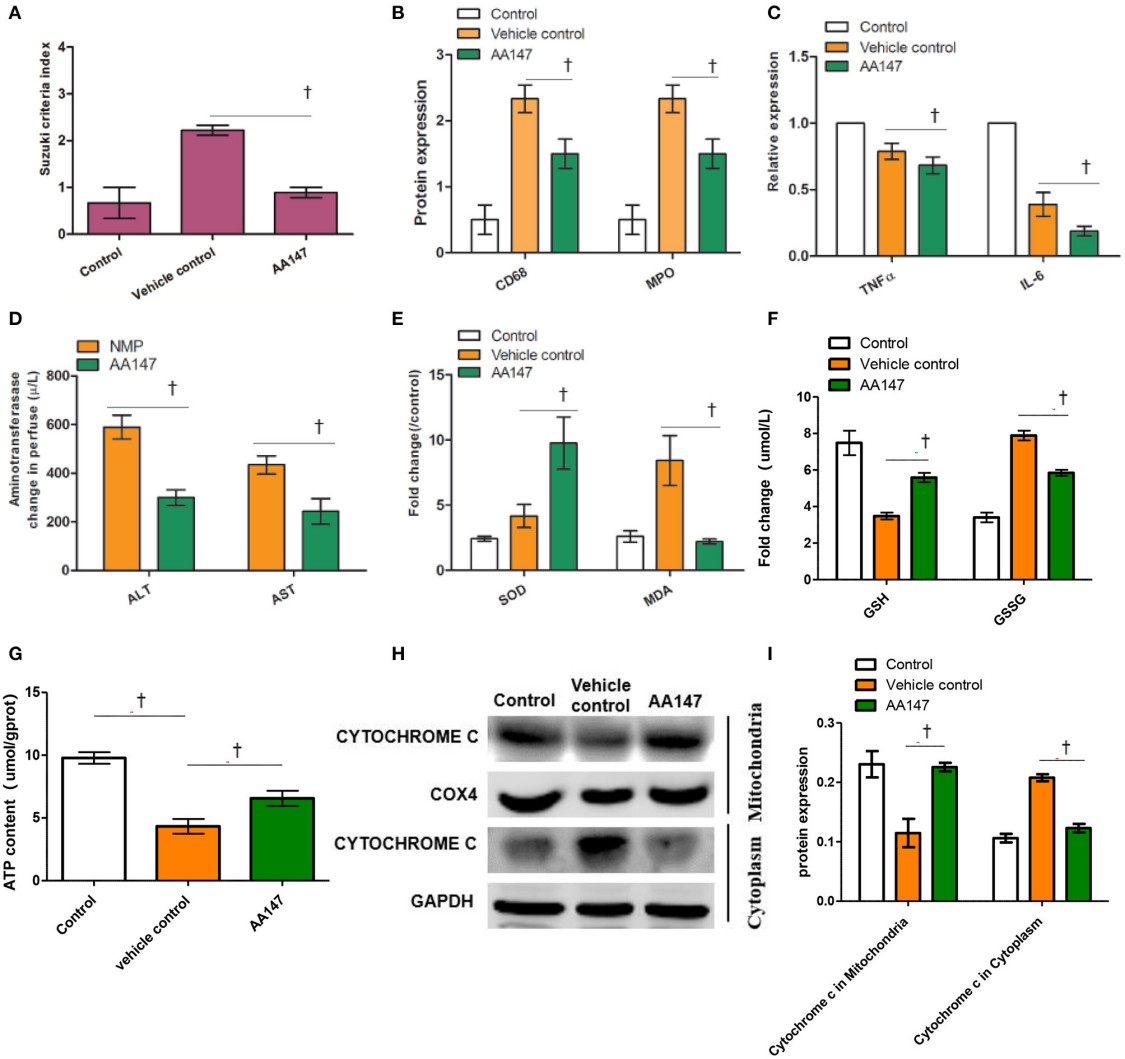
Effect of ATF6 activator (AA147) administration on injury and function of rat livers donation from donation of cardiac death following static cold preservation (SCS) and normothermic machine perfusion (NMP). **(A)** Suzuki sinusoidal injury scores, on a scale from 0 to 4; **(B)** Semi-quantification staining of CD68 and MDA by IHC; **(C)** Relative levels of TNFα and IL-6 in the liver by ELISA; **(D)** Change levels of ALT and AST in perfusate; **(E)** Relative changes of SOD activity and MDA level in the livers; **(F)** Change levels of GSH and GSSG in the liver; **(G)** Relative changes of ATP level in the livers; **(H)** Expression of cytochrome c in mitochondria and in cytoplasm by WB; **(I)** Semi-quantification staining of cytochrome c in mitochondria and in cytoplasm by WB (^†^Denotes significantly different between vehicle control group and AA147 group, *P* < 0.05; *n* = 6).

HE staining showed that warm ischemia, cold preservation, and reperfusion in DCD livers led to pronounced microvesicular steatosis with hepatocyte ballooning, inflammatory cell infiltration, sinusoidal dilatation, and congestion (Supplementary Figure 2). Tissue sections from AA147-treated livers showed limited hepatocyte ballooning and sinusoidal dilatation compared to those in the vehicle control group (Supplementary Figure 2). From HE staining, a significant increase in the Suzuki scores in the livers of the vehicle control group was markedly prevented by AA147 treatment (*t* = 7.000, *P* = 0.002; Figure 4A). Intrahepatic inflammation, indexed by the expression of CD68 and MPO based on IHC, was reduced by AA147 treatment compared to that in the vehicle control group (*t* = 2.712 and 3.354, *P* = 0.022 and 0.007, respectively; Figure 4B). Consistently, levels of pro-inflammatory cytokines, namely TNF-α and IL-6, in the AA147-treated group were 83.900 ± 8.451 pg/mg and 521.554 ± 211.242 pg/mg, whereas in the vehicle control group, they were 99.022 ± 12.100 pg/mg and 1217.328 ± 390.935 pg/mg, respectively, by ELISA, with significant changes noted between the two groups (*t* = 2.510 and 3.835, *P* = 0.031 and 0.003, respectively; Figure 4C).

The functional parameters, ALT and AST levels, are well-defined surrogate markers for liver graft function and survival after transplantation (19, 20). We found that the elevated levels of ALT and AST in perfusate with AA147 following NMP were significantly reduced in comparison to those in the vehicle control group (*t* = 4.925 and 4.064, *P* = 0.008 and 0.015, respectively; Figure 4D). In addition, hepatic SOD activity and MDA levels in AA147-treated group were 9.7 ± 3.5 U/mg and 2.8 ± 0.4 μmol/mg, respectively, which were significantly different than the levels of 4.2 ± 1.5 U/mg and 8.4 ± 3.3 μmol/mg in the vehicle control group (*t* = 2.549 and 3.228, *P* = 0.043 and 0.032, respectively; Figure 4E). GSH, GSSG, and ATP, are widely accepted markers for oxidative stress. We found that the levels of GSH and ATP in perfusate with AA147 following NMP were elevated and GSSG were reduced in comparison to those in the vehicle control group (*t* = 6.524, 5.691, and 3.519, *P* = 0.0029, 0.0047, and 0.012, respectively; Figures 4F,G). Further, we tested the expression of cytochrome c which is an important indicator of mitochondrial damage in the cytoplasm and mitochondria. The results of WB showed that the protein expression of cytochrome c in mitochondria was induced, whereas the protein expression of cytochrome c in cytoplasm was inhibited in rat liver tissues of the AA147-treated group, compared to levels in the vehicle control group (*t* = 4.453 and 9.214, *P* = 0.0056 and 0.0004, respectively; Figures 4H,I). These results indicated that NMP in combination with AA147 could suppress liver inflammation, reduce oxidative stress and improve functions of DCD livers compared to those with NMP alone.

## Discussion

NMP can protect the organ from further ATP depletion and the accumulation of metabolic products during cold ischemia (2). NMP or NRP with D-HOPE for DCD grafts with subsequent transplantation are becoming routine practice in some experienced transplant centers (21–23). Since oxidative tissue injury and activation of the inflammation response during NMP has been demonstrated (2), the ER stress pathway and unfolded protein response might participate in reperfusion and NMP of the DCD liver graft. In this study, we demonstrated that both cytoplasmic and nuclear ATF6 were elevated in DCD livers processed by NMP, indicating that an ATF6-associated pathway could participate in the regulation of liver graft retrieval from DCD. The histological evaluation in the current study and that in previous studies (24) have demonstrated that DCD donors with long-term cold preservation are characterized by both hepatocyte damage and the activation of inflammatory cells. ATF6 might directly alleviate hepatic injury through the cellular adaptive response (8); in addition, ATF6 can mediate the liver pro-inflammatory response through Kupffer cells (7). Therefore, a treatment strategy targeting ER stress-associated mediators was suggested during NMP.

Our study directly evaluated the therapeutic regimen under NMP for DCD livers before transplantation. An ATF6-specific agonist, AA147 (10) which was rarely reported, was used in NMP system, and *ATF6* and the ATF6-regulated genes *PPARA* (16) and *RCAN1* (17) were significantly altered accordingly. As a result, this improved liver retrieval from DCD, indexed by a lower Suzuki score and decreased aminotransferase and MDA levels and GSSG levels and inflammation parameters (IL-6 and TNF-α), as well as higher SOD activity and higher GSH levels and higher ATP levels. These findings indicated hepatocyte protection mediated by ATF6 stimulation through direct and/or indirect effects via inflammation. To our knowledge, this is the first study indicating that an exogenous ATF6 activator could rescue the liver from acute inflammation-associated injury. Therefore, pharmacologic ATF6 activation might have significant clinical utility, together with the NMP system, to improve the function of the DCD liver.

Our current data indicated a critical role for the ATF6 pathway in DCD liver retrieval and suggested a novel approach to improve liver retrieval after cold preservation. However, to avoid microthrombosis from the DCD liver graft, we used the heparinized perfusate with full blood. This might limit the application of comprehensive functional blood tests, which are sensitive to coagulation and hemolysis. Translation of the current findings to the clinic still requires further verification based on liver graft viability and recipient survival after liver transplantation. Although our data showed that an ATF6 activator could reduce the expression of inflammation markers and pro-inflammatory cytokines in the DCD grafts under NMP, it remains unclear whether and how pharmacological ATF6 activation modulates immune responses in DCD livers.

In conclusion, our study showed that intrahepatic ATF6 is elevated and activated in an *ex vivo* NMP model and that an exogenous ATF6 activator confers a functional benefit for DCD livers. Liver NMP after warm ischemia and prolonged cold ischemia might trigger multiple physiologic responses and potentially complicate liver injury. Therefore, treatment modality of the ATF6 pathway provides a promising approach to rescue liver grafts subjected to DCD and prolong cold preservation before transplantation. Pharmacological ATF6 agonist may benefit DCD liver retrieval through NMP system. Further evaluation of the results is indicated.

## Data Availability Statement

The raw data supporting the conclusions of this article will be made available by the authors, without undue reservation.

## Ethics Statement

The animal study was reviewed and approved by Zhengzhou University Committee on Use and Care of Animals (No.2019-KY-019).

## Author Contributions

S-JZ, W-ZG, J-HS, and YJ designed the study. NC and J-HS performed the experiments and drafted the manuscript. NC, Y-BS, H-PZ, S-LC, HY, and X-DL carried out the experiments. J-HS and NC designed the study and revised the manuscript. All authors read and approved the final manuscript.

## Conflict of Interest

The authors declare that the research was conducted in the absence of any commercial or financial relationships that could be construed as a potential conflict of interest.

## Supplementary Material

Supplementary Figure 1 Gross observation of livers from donation of cardiac dead (DCD) rats following static cold preservation (SCS) and normothermic machine perfusion (NMP) **(A)** the DCD liver after 8 h of cold preservation (before NMP); **(B)** the DCD liver after 8 h of cold preservation (before NMP, connecting to the NMP system); **(C)** the DCD liver during the initial stage of NMP; **(D)** the DCD liver after NMP for 2 h.

Supplementary Figure 2 Histology (haematoxylin and eosin staining) and immunostaining of CD68 and MDA (haematoxylin counterstaining). Original magnification ×200, scale bars 50 μm; arrows pointing at hepatocyte ballooning, inflammatory cell infiltration, sinusoidal dilatation, and congestion.
