# Supplementary figures and images for: Pharmacological Activating Transcription Factor 6 Activation Is Beneficial for Liver Retrieval With ex vivo Normothermic Mechanical Perfusion From Cardiac Dead Donor Rats
Source: Front Surg. 2021 Jun 18;8:665260. doi: 10.3389/fsurg.2021.665260 (PMC8249577; doi:10.3389/fsurg.2021.665260)

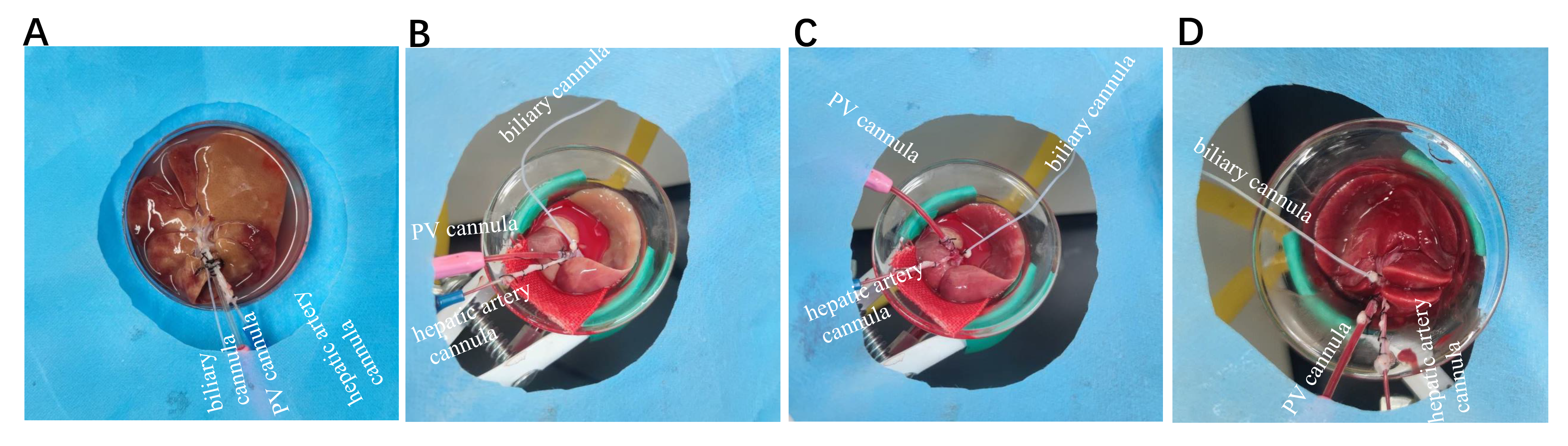

Supplement: Supplementary Figure 1 — Gross observation of livers from donation of cardiac dead (DCD) rats following static cold preservation (SCS) and normothermic machine perfusion (NMP) (A) the DCD liver after 8 h of cold preservation (before NMP); (B) the DCD liver after 8 h of cold preservation (before NMP, connecting to the NMP system); (C) the DCD liver during the initial stage of NMP; (D) the DCD liver after NMP for 2 h. [file Image_1.TIF]

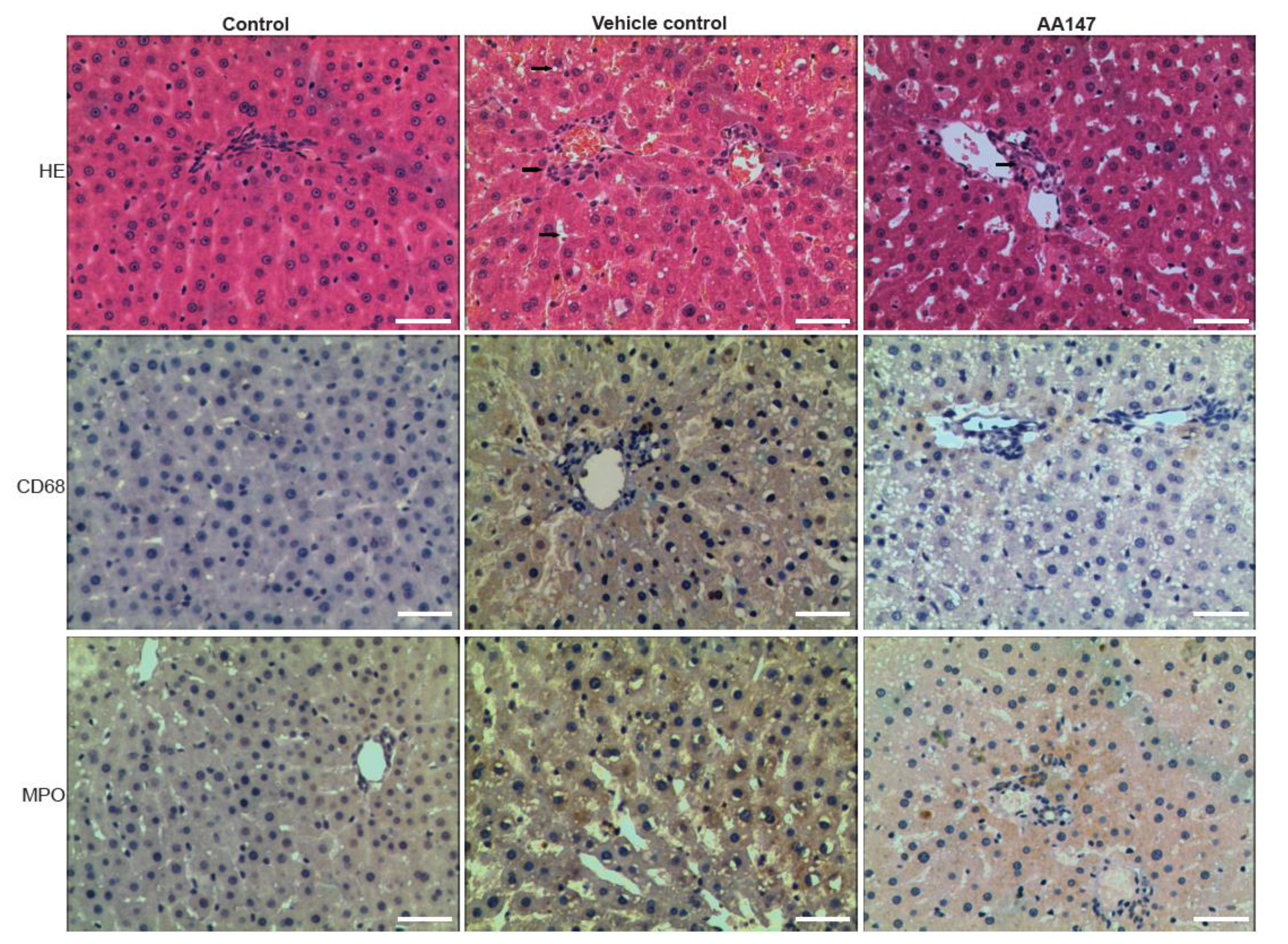

Supplement: Supplementary Figure 2 — Histology (haematoxylin and eosin staining) and immunostaining of CD68 and MDA (haematoxylin counterstaining). Original magnification ×200, scale bars 50 μm; arrows pointing at hepatocyte ballooning, inflammatory cell infiltration, sinusoidal dilatation, and congestion. [file Image_2.TIF]
